# Gerstmann-Sträussler-Scheinker syndrome with unexpected concomitant *GRN* variant: case report

**DOI:** 10.3389/fnins.2026.1857360

**Published:** 2026-07-13

**Authors:** Matyas Sykora, Bibiana Krenkova, Eva Parobkova, Jiří Keller, Svatopluk Ostry, Robert Rusina, Radoslav Matej

**Affiliations:** 1 Department of Pathology and Molecular Medicine, Third Faculty of Medicine, Charles University and Thomayer University Hospital, Prague, Czechia; 2 Department of Neurology, Third Faculty of Medicine, Charles University and Thomayer University Hospital, Prague, Czechia; 3 BrainBank, Third Faculty of Medicine, Charles University and Thomayer University Hospital, Prague, Czechia; 4 Department of Neurology, Hospital Ceske Budejovice, Ceske Budejovice, Czechia; 5 Department of Radiology, University Hospital Motol and Homolka, Prague, Czechia; 6 Department of Neurology, Third Faculty of Medicine, Charles University, Prague, Czechia; 7 Department of Neurosurgery and Neurooncology, First Faculty of Medicine, Charles University in Prague and Military University Hospital, Prague, Czechia; 8 Department of Neurology University Hospital Hradec Kralove, Faculty of Medicine Hradec Kralove, Charles University, Hradec Kralove, Czechia; 9 Department of Pathology, First Faculty of Medicine, Charles University and General University Hospital, Prague, Czechia; 10 Department of Pathology, Third Faculty of Medicine, Charles University and University Hospital Kralovske Vinohrady, Prague, Czechia

**Keywords:** case report, Gerstmann-Sträussler-Scheinker syndrome, prion, progranulin gene, *TMEM106B*, variant

## Abstract

The objective is to report a patient with Gerstmann-Sträussler-Scheinker syndrome caused by a pathogenic *PRNP* P102L variant harboring an unexpected concomitant pathogenic *GRN* variant p.R110X and to discuss the potential contribution of combined genetic pathology to the clinical and neuroimaging phenotype confirmed by autopsy. Moreover, we discuss the potential role of *TMEM106B* as an important modifier of the protein TDP-43 neuropathology associated with the *GRN* mutation in this case. The patient underwent detailed clinical assessment, serial neuropsychological evaluation, brain MRI, cerebrospinal fluid analysis, whole-exome sequencing, and next generation sequencing. A postmortem neuropathologic examination was performed to confirm the diagnosis. The patient presented slowly progressive paresthesia, cerebellar ataxia, dysarthria, and later cognitive and behavioral changes. Genetic testing revealed a heterozygous *PRNP* P102L variant and an unpenetrated *GRN* p.R110X variant; a protective *TMEM106B* polymorphism associated with TDP-43 pathology was also identified. Neuroimaging demonstrated progressive cerebellar and parietal atrophy with asymmetric left frontal opercular and insular involvement. The clinical course was dominated by a cerebellar GSS phenotype. The patient died 4 years after symptom onset. Neuropathology confirmed GSS, nevertheless without detectable TDP-43-associated neuropathology. This case highlights the diagnostic complexity of rare neurodegenerative disorders and illustrates that pathogenic variants may not influence phenotypic expression. Comprehensive genetic testing should be considered in atypical cases, as certain genetic variants may contribute to phenotypic variability and represent potential modifiers of phenotypic expression.

## Introduction

Gerstmann-Sträussler-Scheinker syndrome (GSS) is a rare inherited human prion disorder caused mostly by the P102L variant in the prion protein-encoding gene (*PRNP*), with autosomal dominant transmission (Chen et al., 2024) and an estimated prevalence of 1–10 out of 100,000,000 worldwide (Chen and Dong, 2016), although its prevalence may be even higher depending on endemic and familiar conditions (Tesar et al., 2019). GSS is often associated with severe spongiform vacuolation and loss of neurons (Baldwin and Correll, 2019; Shin et al., 2023). Clinical symptoms mostly occur between the ages of 20 and 80, and survival ranges from a few months to 10 years (Baldwin and Correll, 2019).

Four clinical subtypes of P102L GSS have been identified: typical GSS; GSS with areflexia and paresthesia; Creutzfeldt-Jakob-disease-like phenotype; and pure dementia GSS (Tesar et al., 2019).

Considerable clinical variability even within the same family can be observed (Chen et al., 2024; Bugiani et al., 2000). Cerebrospinal fluid 14-3-3 positivity and high total tau protein levels are often not sensitive enough to detect GSS; nevertheless, even RT-QuIC can yield false negative results. MRI can show generalized atrophy of brain cortices or the cerebellum; however, early in the disease course can be normal (Tesar et al., 2019; Yoshimura et al., 2018).

Progranulin (PGRN), a protein encoded by *GRN* gene, plays an important role in microglial function, neuroinflammatory regulation, and lysosomal homeostasis. GRN deficiency has been associated with impaired lysosomal function and altered protein degradation pathways, contributing to TDP-43 accumulation patology (Sung et al., 2024).

Variants in the progranulin gene (*GRN*) are associated with a various spectrum of phenotypes of frontotemporal lobar degeneration (FTLD): behavioral variant of frontotemporal dementia (bvFTD), semantic variant of primary progressive aphasia (svPPA), corticobasal syndrome, or posterior cortical atrophy (Caroppo et al., 2015; Le Ber et al., 2008). In *GRN* carriers, atrophy predominantly involves the frontal, temporal, and parietal cortices but preserves the occipital regions at onset (Whitwell et al., 2012). *GRN* variants are associated with TDP-43 neuronal inclusions (Sung et al., 2024; Neumann et al., 2006).

*TMEM106B* is considered an important genetic modifier in GRN-associated disorders through its influence on lysosomal morphology and function. Moreover, TMEM106B-mediated modulation of lysosomal pathways has been proposed as a potential mechanism influencing susceptibility to TDP-43 neuropathology (Pottier et al., 2018; Mirza et al., 2025; Perneel et al., 2023).

We report a patient with a unique co-occurrence of two rare pathogenic variants, the P102L variant in *PRNP* and the p.R110X variant in *GRN* and protective TMEM106B allele.

## Case description

A 46-year-old woman with an unclear family history of a non-specified “neurological” disorder leading to death at the age of 50 years in her paternal grandmother presented with widespread paresthesia and dizziness of unremarkable onset, and cinnarizine and betahistine were administered. Early on, dysarthria and alexia with gait instability and lower limb ataxia ensued, and the patient was admitted to a regional hospital. Later, she experienced depressive symptoms.

Initial brain and cervical spine MRIs yielded no significant abnormalities, and only mild cerebellar atrophy and parietal atrophy were present (Figures 1a, b). Laboratory examinations ruled out secondary etiology; CSF analysis was normal. Neuropsychological profiling proved mild cognitive decline, particularly in working memory and attention, which initially seemed linked to the depressive syndrome treated with citalopram. Hereditary ataxia testing was negative.

**Figure 1:**
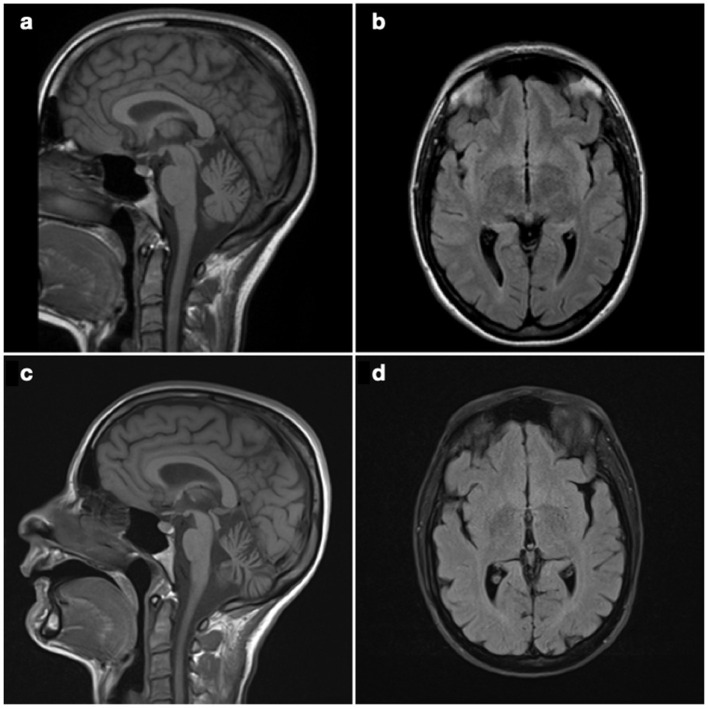
Brain MRI progression over time: Initial sequences **(a, b)** were obtained at Ceské Budějovice Hospital in 2017 and showed no remarkable pathological changes. Follow-up MRI performed at Thomayer University Hospital in Prague in 2021 **(c, d)** demonstrated increased cerebellar atrophy (mainly involving the vermis) and parietal atrophy on FLAIR sequences. In addition, discrete asymmetric atrophy of the frontal operculum and insular cortex, predominantly on the left side, was observed.

Due to suspected atypical neurodegeneration, the patient was referred to a university hospital. Genetic analysis (whole exome sequencing) revealed the P102L variant in the *PRNP* gene and, surprisingly, a concomitant p.R110X variant in the *GRN* gene. The *PRNP* p.P102L variant represents a classic pathogenic alteration associated with Gerstmann-Sträussler-Scheinker syndrome (GSS). At the molecular level, it leads to conformational instability of the prion protein (PrP), promoting its misfolding and conversion to the pathological PrP^∧^Sc isoform. The subsequent accumulation of pathological PrP leads to the formation of amyloid deposits and progressive neurodegeneration. P102L is associated with significant phenotypic and neuropathological variability, which may be related to different conformations of prion strains and biochemical properties of PrP fragments. The clinical diagnosis was GSS (subtype of paresthesia with areflexia) (Tesar et al., 2019), which progressively worsened into a major cerebellar syndrome with severe ataxia.

The patient was consulted with a prion specialist to provide best supportive care. The clinical presentation further worsened toward severe dysarthria with saccadic speech, horizontal and vertical nystagmus, gaze apraxia with difficulties in gaze fixation to the target, limb ataxia with hypermetria and areflexia on the lower extremities. 2 years after disease onset, symptoms resulted in dependence on family members. Pregabalin was prescribed for widespread paresthesia. Amantadine was administered off-label to address gait impairment. All medications were administered in maximal doses with good tolerance and adherence, nevertheless without significant clinical impact. Supportive therapy included physiotherapy and speech therapy and palliative care. Neuropsychology testing concluded considerable impairment in visuospatial processing and language.

To a lesser degree, dysexecutive syndrome with disinhibition and emotional lability was present. EEG did not reveal periodic patterns. Brain MRI showed increased cerebellar (mainly of the vermis) and parietal atrophy and asymmetrical atrophy of the frontal operculum and insular cortex predominantly on the left side (Figures 1c, d).

The prognosis of the patient was poor due to a lethal diagnosis, and the patient died 4 years after disease onset from brain edema and intercurrent infection. Autopsy confirmed typical neuropathology of GSS in line with the previously detected *PRNP* gene sequence variant P102L (c.305C>T) (CCG>CTG) in heterozygous form overlapping with the known p.R110X variant in the *GRN* gene sequence. *PRNP* p.P102L: classified as a pathogenic variant, in accordance with the literature in Gerstmann-Sträussler-Scheinker syndrome (GSS). The *GRN* p.R110X variant has already been reported in public genetic databases (including NCBI/ClinVar) as a pathogenic or likely pathogenic loss-of-function variant associated with frontotemporal lobar degeneration (FTLD) (Chen-Plotkin et al., 2011). Both variants were confirmed by Sanger sequencing. Severe generalized neuronal atrophy with prominent astrogliosis was observed in the cerebellum and subcortical gray matter; however, these changes combined with the pattern of typical spongiform encephalopathy were also observed in all cortical regions. Moreover, characteristic “kuru-like” prion protein plaques focally forming multicentric plaques were seen either in standard H&E staining or were clearly visualized by immunohistochemical reaction with antibodies raised against prion protein (clones 12F10 and 3F4). Surprisingly, neuropathology focused on pathological depositions of TDP-43 protein, a typical hallmark of the previously reported pathogenic *GRN* variant p.R110X, was negative in all investigated areas, including brain structures suspected of involvement from MRI scans. Only unspecific “dash-like” and “dot-like” intraneuronal depositions were observed in immunohistochemical investigation using antibodies against TDP-43 and its hyperphosphorylated form in the amygdala and hippocampal regions without pathognomonic diagnostic protein inclusions, typically observed in *GRN* mutations cases. Additionally, there were incidental findings of initial tauopathies, predominantly picture of primary age-related tauopathy (PART) and argyrophilic grain disease (AGD), although any pathogenic aberration was recorded in the *MAPT* gene.

The detailed course of the disease is summarized in Figure 2. The family pedigree is summarized in Figure 3. The patient's mother was genetically tested negative for both *GRN* and *PRNP* gene variants. The patient's father died from malignancy 10 years ago, and no tissue samples for DNA testing were available.

**Figure 2:**
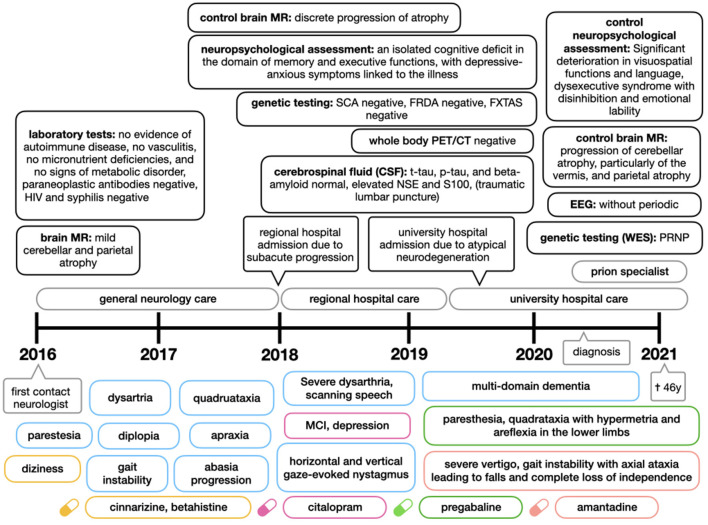
Detailed timeline of the disease course, highlighting key clinical events, diagnostic findings, and therapeutic interventions. Above the timeline, major medical events, examinations, and relevant results are arranged in approximate chronological order. Below the timeline, the gradual onset and progression of symptoms are depicted in temporal sequence, with therapeutic interventions indicated by color coding. All medications were administered at the maximum approved doses and were well tolerated with good adherence; however, no clinically meaningful improvement in symptoms was observed.

**Figure 3:**
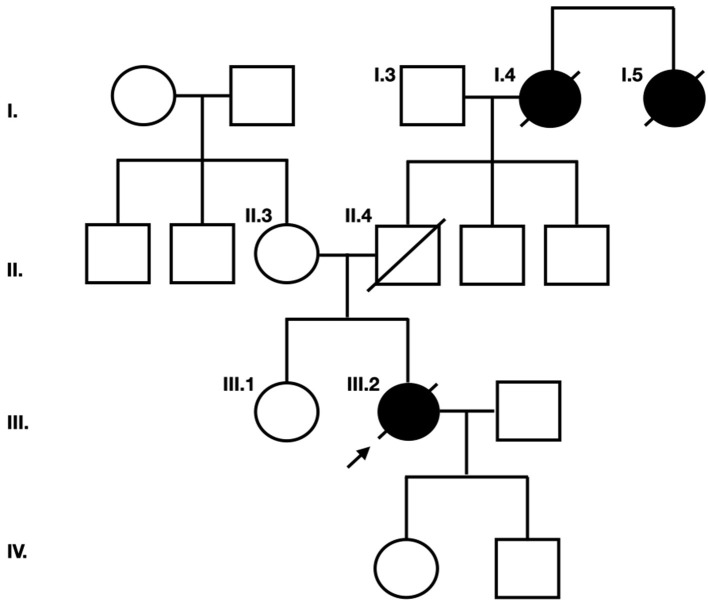
The proband's paternal grandmother **(I.4)** had unspecified early-onset dementia, with death at the age of 50. Furthermore, archival records indicate that her sister **(I.5)** had neuropathologically confirmed GSS. However, due to a fire that destroyed the original samples, the exact sequence of the pathogenetic variant cannot be confirmed. The proband's father **(II.4)** died in mid-life from colorectal carcinoma, and another limitation is the inability to analyze his tissue samples, as they have been disposed from the archive. The mother **(III.1)** has negative genetic testing for *PRNP* and *GRN* variants and no personal or family history of dementia, which further supports the paternal origin of the variant. We negatively tested the proband's sister **(III.1)**. The last generation of the family is asymptomatic and declined genetic testing **(IV)**.

## Discussion

We present a unique case with a concomitant presence of two distinct rare variants. The *PRNP* variant in our case is presumed to be of paternal origin. Retrospective family history revealed that the proband's paternal grandmother (I.4) had unspecified early-onset dementia, with death at the age of 50. Furthermore, archival records indicate that her sister (I.5) had neuropathologically confirmed GSS. However, due to a fire that destroyed the original samples, the exact sequence of the pathogenetic variant cannot be confirmed. The proband's father (II.4) died in mid-life from colorectal carcinoma, and another limitation is the inability to analyze his tissue samples, as they have been disposed from the archive. The mother (III.1) has negative genetic testing for *PRNP* and *GRN* variants and no personal or family history of dementia, which further supports the paternal origin of the variant. We negatively tested the proband's sister (III.1); nevertheless, the origin of the *GRN* variant remains unclear due to the absence of any biological material from other relatives.

A non-specific clinical presentation posed a significant diagnostic challenge. The clinical presentation of a very slow onset of paresthesia with cerebellar symptoms together with marked cerebellar and brainstem atrophy and the P102L variant in the *PRNP* gene was clearly compatible with GSS [subtype GSS with paresthesia and areflexia (Tesar et al., 2019)]. The cognitive profile was compatible with GSS as well; early manifestations included prodromal depression, disinhibition, and emotional lability. In later stages, significant deficits in working memory and attention developed, and impairments in visuospatial processing and language appeared to be associated with a prevalent dysexecutive syndrome.

The impact of the *GRN* variant on the phenotype is more questionable. Different clinical phenotypes have been linked with *GRN* variants, such as bvFTD (Baker et al., 2006), svPPA (Samra et al., 2023), and rarely corticobasal syndrome or posterior cortical atrophy (Caroppo et al., 2015; Giunta et al., 2021). Moreover, autoptic investigation does not reveal typical morphological changes previously described in patients with *GRN* variants. A possible driving mechanism for this finding may be the modifier effect of *TMEM106B* in *GRN*-associated protein TDP-43 neuropathology through its influence on lysosomal morphology and function, which was proposed as a potential mechanism modulating susceptibility to TDP-43 protein inclusion-associated neuropathology (Pottier et al., 2018; Mirza et al., 2025; Perneel et al., 2023; Takahashi and Strittmatter, 2025).

We thus hypothesize that in our case, the allele in the *TMEM106B* gene, rs3173615 (p.Thr185Ser), might have impacted on the absence of TDP-43 inclusions in the brain tissue expected as result of the *GRN* mutation. According to the literature, *TMEM106B* gene polymorphism is associated with reduced symptom severity and several clinical studies have since been published (Pottier et al., 2018); Nicholson and Rademakers stated that significant number of individuals with pathogenic *GRN* mutations and two protective *TMEM106B* alleles may remain without symptoms throughout life (Nicholson and Rademakers, 2016). Vandebergh et al. (2024) identified associations of *TMEM106B* in the *GRN* and *C9orf72* genetic groups, in line with *TMEM106B* being identified as a important phenotypic modifier in those with TDP-43 pathology. Similar to our observation, in a recently published *GRN* family with a proband presenting with young-onset FTD whose parent is an asymptomatic carrier in the 1980s, the family may be protected from developing disease symptoms because of the modifying effect of *TMEM106B* (Perneel et al., 2023).

Variants in the *TMEM106B* gene have been proposed as modifiers of FTD disease risk, especially in *GRN* mutation carriers, due to their modifying effects on biomarkers, imaging, cognitive and clinical outcomes in genetic FTD and modifying the effect of autosomal dominant FTD mutations on the natural course of genetic FTD, particularly TDP-43 pathology (Mirza et al., 2025). Moreover, Feng et al. suggest that modulation of *TMEM106B* levels might affect disease progression of FTLD patients with *GRN* mutations in their review (Feng et al., 2021).

We acknowledge that our study has several limitations. The hypothesized protective effect of the *TMEM106B* variants on other genes involved in neurodegeneration still needs more evidence. Another plausible explanation for the absence of TDP-43 neuropathology in brain tissue could be the relatively short disease course, with the possibility that the patient died before the neuropathological consequences associated with the *GRN* variant became fully manifest. An additional limitation is that, although *GRN* loss-of-function variants are generally associated with high age-dependent penetrance, nevertheless variant-specific penetrance data for p.R110X remain limited. A further limitation is the absence of relevant biological material to confirm the origin of both variants. Although we excluded maternal origin and the family history was positive in the paternal pedigree, we cannot confirm a direct transmission of both pathogenic variants. Moreover, there is lack of therapeutic recommendations; all therapeutic approaches remain supportive or off-label.

In conclusion, atypical neurodegenerative disorders may occur in unexpected comorbidity, and clinical manifestations may reach a broad spectrum of phenotypes according to the underlying neuropathology. Our case demonstrates an intriguing co-expression of three different genetic variants implied in neurodegeneration (*PRNP, GRN, TMEM106B*), largely predominated by the *PRNP* mutation resulting in fully developed GSS, clinically and neuropathologically, and, moreover, the surprising absence of TDP-43 protein inclusions in brain tissue expected from the pathogenic *GRN* mutation. Likely pathogenic and pathogenic variants as well as potential indirect genetic modifier may indirectly influence expression of pathogenic protein deposits, as in our case, with a possibly protective effect of a *TMEM106B* variant on a *GRN* mutation carrier. Nevertheless, further evidence is needed to support this hypothesis.

## Data Availability

The data supporting the findings of this study are available from the corresponding author upon reasonable request. Due to privacy and ethical restrictions, the data are not publicly available.
